# Pathogenesis of FUS-associated ALS and FTD: insights from rodent models

**DOI:** 10.1186/s40478-016-0358-8

**Published:** 2016-09-06

**Authors:** Matthew Nolan, Kevin Talbot, Olaf Ansorge

**Affiliations:** 1 Neuropathology, Nuffield Department of Clinical Neurosciences, University of Oxford, Oxford, UK; 2 Nuffield Department of Clinical Neurosciences, University of Oxford, Oxford, UK

**Keywords:** Amyotrophic lateral sclerosis, Frontotemporal dementia, MND, Frontotemporal lobar degeneration, FUS, FUSopathy, TDP-43

## Abstract

Disruptions to genes linked to RNA processing and homeostasis are implicated in the pathogenesis of two pathologically related but clinically heterogeneous neurodegenerative diseases, amyotrophic lateral sclerosis (ALS) and frontotemporal dementia (FTD). Mutations in the Fused-in-Sarcoma (*FUS*) gene encoding a 526 amino-acid RNA-binding protein are found in a small subset of ALS cases, but *FUS* mutations do not appear to be a direct cause of FTD. Structural and functional similarities between FUS and another ALS-related RNA-binding protein, TDP-43, highlight the potential importance of aberrant RNA processing in ALS/FTD, and this pathway is now a major focus of interest. Recently, several research groups have reported transgenic vertebrate models of FUSopathy, with varying results. Here, we discuss the evidence for *FUS* pathogenicity in ALS/FTD, review the experimental approaches used and phenotypic features of *FUS* rodent models reported to date, and outline their contribution to our understanding of pathogenic mechanisms. Further refinement of vertebrate models will likely aid our understanding of the role of *FUS* in both diseases.

## Introduction

Neurodegenerative diseases such as amyotrophic lateral sclerosis (ALS) and frontotemporal dementia (FTD) are characterised by the progressive destruction of neurons, associated with the aggregation and deposition of one or more types of proteinaceous inclusion. ALS is characterised by the degeneration of both upper (UMN) and lower (LMN) motor neurons, causing muscular atrophy and progressive paralysis [2], with death usually occurring within 3 years of symptom onset [1]. The majority of cases are classified as sporadic (sALS), with no obvious Mendelian inherited component. However, around 10 % of cases are caused by mutations in one or more known ALS genes [2] both in patients where there is a clear family history (familial ALS, fALS), but also in a minority of sALS cases, in which the mutation acts as a rare variant of significant disease determining effect. Treatment for ALS is mainly supportive, involving physical therapy, nutritional support and artificial ventilation in the later stages [10]. While widely prescribed, riluzole, the only pharmacological treatment available, produces only modest increases in survival in clinical trials [83].

FTD and its pathological presentation - Frontotemporal Lobar Degeneration (FTLD) – is the second most common form of young-onset dementia after Alzheimer’s disease [25]. FTLD is characterised by widespread degeneration of neurons in the frontal and temporal lobes, presenting clinically as significant behavioural or language abnormalities [7], with relative sparing of memory until late disease stages. FTD is clinically categorised as one of three subtypes: behavioural variant FTD (bvFTD), semantic dementia (SD) or progressive non-fluent aphasia (PNFA) [51, 64]. As for ALS, no disease modifying therapy is available. Symptomatic treatment includes selective serotonin re-uptake inhibitors (SSRI’s) to control compulsive behaviour [59] and general supportive care [7]. Diagnosis for both ALS and FTD is made primarily on clinical grounds in the context of an appropriate history and examination. Neurophysiological testing is useful in ALS, but other investigations such as imaging are essentially used to rule out mimic disorders [26, 72, 84]. While detectable cognitive dysfunction may be present in up to 50 % of ALS cases [58, 78], around 15 % of patients meet formal clinical criteria for both ALS and FTD (ALS-FTD) [56, 58, 68], the combination of which being associated with a worse prognosis and reduction in survival time of around one year [56].

Despite significant clinical heterogeneity, the overlap in genetics and pathology between ALS and FTD have led them to be widely considered as being part of a clinico-pathological disease continuum, with pure ALS and pure FTD representing spectral extremes [28, 89]. Cases with mutations in more than one ALS/FTD gene are being increasingly reported [14, 36, 87], suggesting that ‘oligogenic’ factors may be one element in a ‘multiple-hit’ model of disease [86]. In 2009, mutations to the Fused-in-Sarcoma (*FUS*) gene were identified as causative in a small number of ALS cases [39, 90]. This review summarises our understanding of the genetic and neuropathological features of *FUS*-related ALS/FTD, and critically appraises the progress that has been made in modelling *FUS* mutations in-vivo, with a particular focus on rodent models.

### Fused-in-Sarcoma (*FUS*)

The most frequent genetic mutation linking ALS and FTD is a hexanucleotide (G_4_C_2_) repeat expansion within an intronic promoter region of the *C9ORF72* gene [20, 66], accounting for around 35 % fALS cases [65] and around 25 % FTD cases [85]. Transactive DNA-binding protein 43 kDa (TDP-43) is the major component of inclusions in motor neurons of sALS and some cases of FTLD [4, 54], and mutations to its corresponding gene, *TARDBP*, are responsible for a small number of both fALS and sALS cases [27, 75], as well as FTD. Mutations to the Fused-in-Sarcoma (*FUS)* gene on chromosome 16 are responsible for a small, but important subset of both familial and sporadic ALS [39, 90] accounting for around 4 and 1 % of total cases respectively [21, 45]. Interestingly, variants have also been implicated in essential tremor [21].

**Fig. 1:**
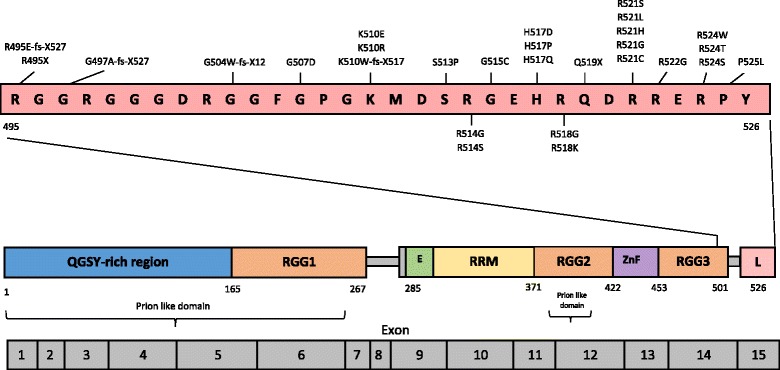
Structure and functional domains of FUS. FUS is a member of the TET family of proteins, and contains several functional domains including a QGSY-rich region, multiple RNA binding regions, a C-terminal Zinc-finger motif and two putative ‘prion-like’ domains. The majority of mutations in ALS-*FUS* are located within the C-terminal nuclear localization signal domain in exon 15. Figure adapted by author from Vance et al. [91] and Deng et al. [21]

*FUS* encodes a 526 amino acid, 15-exon RNA binding protein of the FET family, containing several distinct functional domains including a RNA-recognition motif and a highly-conserved C-terminal nuclear localization signal (NLS) [21] (Fig. 1), where many of the identified mutations occur. This domain architecture is shared with Ewing’s Sarcoma (EWS) protein and TATA-binding protein-associated factor 15 (TAF15), which together with FUS are referred to as the FET family of proteins that were initially characterised as part of fusion oncogenes in human malignancies [3]. The precise normal physiological function of *FUS* is unclear. However, known roles include transcriptional control [79], RNA processing through splicing regulation of pre-mRNA’s [40], and DNA repair [93, 46]. Recently, *FUS* mutations have been shown to significantly alter target gene expression by binding target gene mRNA within the aggregates of transfected human cells [16]. While there is still some debate on the nature of FUS toxicity, the range of functions involving *FUS* highlight its potential susceptibility to dysfunction and the consequences for the maintenance of cellular RNA homeostasis. Evidence to support both gain and loss-of-function mechanisms now exists, and it appears likely that both mechanisms are implicated, depending on the particular mutation and its functional connotations.

Under normal physiological conditions FUS is predominantly localized in the nucleus in neurons, but is exclusively nuclear-based in glia [3]. However as an RNA-binding protein, it possesses the ability to move between both through its role in nucleocytoplasmic transport [13]. The characteristic presence of FUS-immunoreactive inclusions in the cytoplasm of ALS-*FUS* and FTLD-FUS has led to the suggestion that mislocalization of FUS to the cytoplasm contributes to neurodegeneration in these cases, by a gain-of-toxicity mechanism. This concept is closely tied to the formation of stress granules, which notably contain mutant FUS but not endogenous wild-type FUS [12]. The role of mutant FUS in stress-assembly dynamics is now well documented [11, 12, 18] and illustrates an obvious differential between normal and disease physiology. For example, one study has demonstrated how oxidative stress recruits mutant FUS into stress granules where it can sequester wild-type FUS to disrupt RNA processing and potentially initiate cell death [91]. Knock-down zebrafish models, however, display a subtle motor phenotype and hyper extended axonal branching that cannot be rescued with mutant FUS, suggesting loss-of-function [34], and a combination of both mechanisms remains possible.

### ALS-*FUS*

Over 50 mutations in the *FUS* gene have now been reported in ALS – most of which are mis-sense – with a minority being in-frame deletions [21]. Many, including the most common *FUS* mutation in humans, R521C, occur within the highly conserved C-terminal nuclear localization signal [62]. Nearly all display an autosomal dominant pattern of inheritance, albeit with varying degrees of penetrance. Notably, some mutations such as P525L are associated with a more severe disease progression [31] and juvenile onset [8, 48], with apparently sporadic occurrence, presumably because the condition is frequently lethal before it can be transmitted [35].

**Fig. 2:**
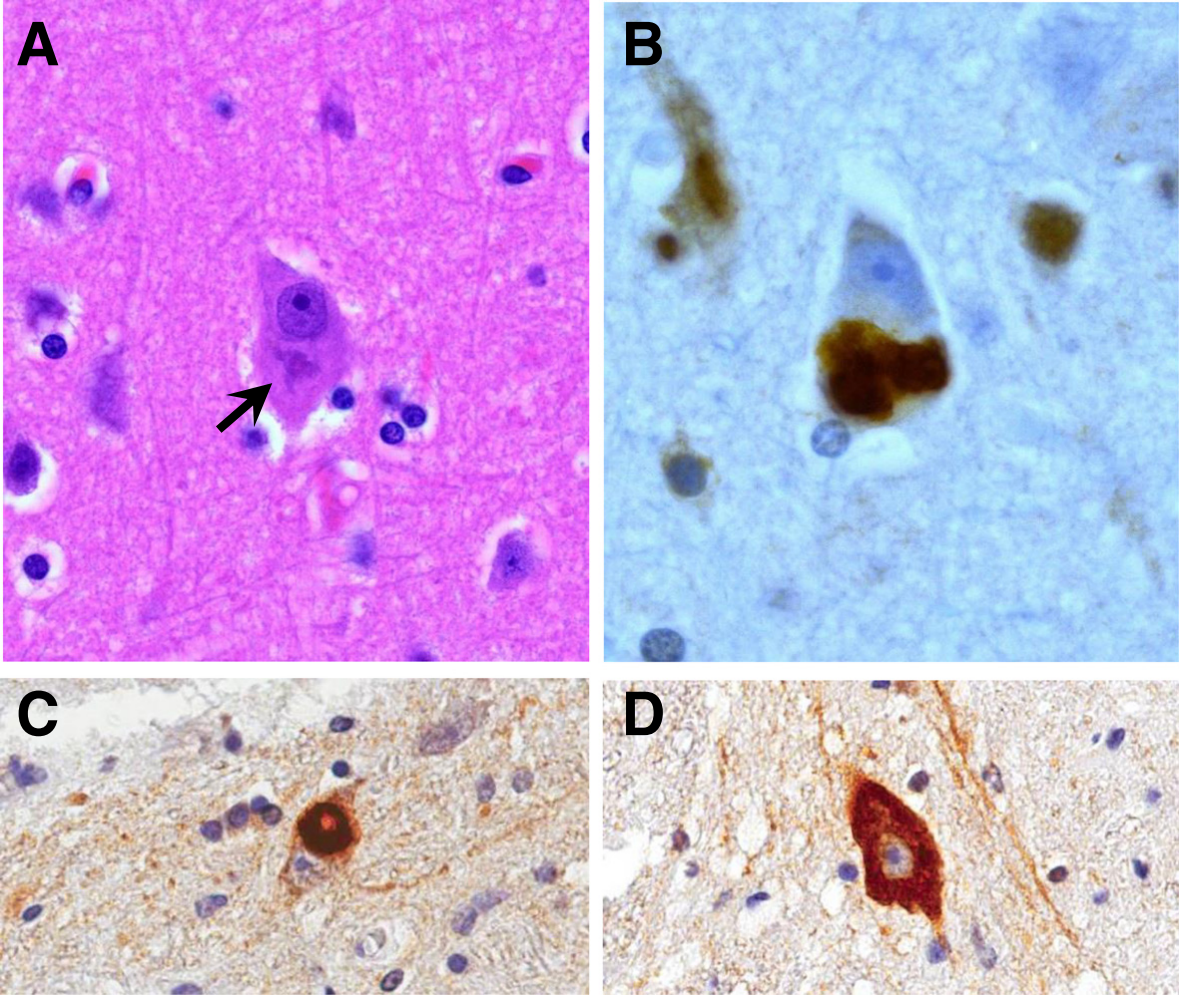
Neuronal and glial cytoplasmic inclusions immunoreactive for FUS define the pathology of both ALS-*FUS* and FTLD-FUS. Basophilic inclusions are present in neurons in ALS-*FUS* (arrowed) and can be viewed using H&E stain, X400 (**a**). Discrete neuronal inclusion immunoreactive for FUS associated with the P525L mutation, X400 (**b**). ALS-*FUS* inclusions in the anterior horn of spinal cord, both X40 Obj (**c**, **d**). Well defined, compact inclusions (**c**) or intense diffuse cytoplasmic staining (**d**) are commonly seen, often with nuclear clearance

The neuropathology of ALS-*FUS* may be related to its specific genetic cause and subsequent disease course [42]. Predominantly a LMN disease with a younger average age of onset and an aggressive course, patients typically display severe neuronal loss in the spinal cord anterior horn with only low or moderate neuronal loss of Betz cells within layer V of the motor cortex [65] (Table 1). Neuronal and glial cytoplasmic inclusions (NCI) containing ubiquitinated FUS in the motor cortex, basal ganglia and spinal cord, as well as dystrophic neurites [2] are seen. TDP-43 pathology is entirely absent. Certain variants including P525L seemingly predispose the formation of basophilic inclusions (BI) [42], which can be readily viewed using haematoxylin and eosin (H&E) (Fig. 2). Clinical presentation is consistent with classical ALS, although three groups have reported similar clinical features in patients with the p.R521C variant, suggesting correlation between individual mutations and specific clinical abnormalities [17, 62, 81].

**Table 1:** Phenotype-pathology correlations summary of FUS-linked human ALS/FTD

Disease		Neuropathology	Genetics	Epidemiology	Clinical features
ALS-FUS		- Degeneration of both upper and lower motor neurons - Significant neuronal loss within anterior horn of spinal cord - Moderate neuronal loss of Betz cells within layer V of motor cortex and motor nuclei of brainstem - Dystrophic neurites, astrogliosis, microglial activation - TDP43-negative, FUS-positive neuronal cytoplasmic inclusions	*FUS* – over 50 mutations described, particularly missense variations within C-terminal Nuclear Localization Signal	*FUS* mutations account for ~4 % fALS and ~1 % sALS	- Progressive muscular atrophy - Dysphagia - Dysarthria - Respiratory - Rigid spasticity - Death normally within 2–3 years from symptom onset
FTLD-FUS	Atypical FTLD with Ub (aFTLD-U)	- Widespread degeneration of frontal cortex and ventral temporal lobe - Tau/TDP43-negative, FUS-positive neuronal or glial inclusions predominantly within hippocampus, amygdala, frontotemporal cortex and striatum	Rare cases of *FUS* variants in clinical FTLD (not confirmed pathologically)	~10 % FTLD cases display FUS pathology	- Normally behavioural variant FTD - Changes in personality and emotion - Irrationality, compulsiveness, confusion, repetition, inappropriate behaviour - Psychiatric symptoms, depression and anxiety common - Memory, motor function and perception are relatively preserved until late disease stages
	NIFID	- Neuronal cytoplasmic inclusions containing abnormal intermediate filament accumulation			
	BIBD	- Significant FUS-pathology plus subcortical basophilic inclusions on H&E staining			

*ALS* Amyotrophic Lateral Sclerosis (familial and sporadic), *FTLD* Frontotemporal Lobar Degeneration, *NIFID* Neuronal Intermediate Filament Inclusion Disease, *BIBD* Basophilic Inclusion Body Disease, *FUS*, Fused-insarcoma; H&E, Haematoxylin and eosin

### FTLD-FUS

*FUS* mutations have only rarely been reported in FTD, and mostly co-exist with ALS, making their significance in its pathogenesis unclear [21, 45, 52, 74]. One study identified a unique variant within exon 4 (P106L) in a patient with pure bvFTD [32] (which could not be confirmed as co-segregating because of lack of DNA from other family members), and another reported a novel mis-sense variation (M254V) with predicted pathogenicity in an FTD patient without ALS [88], but neither case has been confirmed post-mortem. Indeed, there have been no reported cases of pure, clinical FTD that were both genetically and neuropathologically confirmed as *FUS*-related. Additionally, the largest genome-wide association study (GWAS) of clinical FTD to date involving exome sequencing of 3526 FTD patients and 9402 healthy controls found only weak association with variants at the *FUS* locus, with none being at a genome-wide significance level [25]. Genome-wide association studies, however, are only powered to detect an association between common variants and a disease. The picture emerging from ALS genetics, though as yet unconfirmed in FTD, is a genetic contribution mostly accounted for by rare variants.

Despite the absence of confirmed genetic cases, inclusions immunoreactive for FUS are present in a small proportion FTLD cases (FTLD-FUS) and can be neuropathologically sub-categorised as atypical FTLD-U, neuronal intermediate filament inclusion disease (NIFID), or basophilic inclusion body disease (BIBD) [44, 94] (Table 1) . All are defined by the presence of FUS-positive, tau/TDP-43 negative inclusions of varying formation, often co-localized with the other two FET proteins EWS and TAF15, alongside prominent degeneration of the frontal and temporal lobes. Atypical FTLD-U cases display uniform, round neuronal cytoplasmic inclusions (NCI) throughout the brain, but mainly within frontal and temporal neocortex, hippocampus and striatum [43]. NIFID is characterized by NCI’s that are immunoreactive for all class IV neurofilament chains - the form of which vary according to neuronal type and region [43] - with additional cytoplasmic granules of FUS aggregation [53]. BIBD cases show large, round basophilic neuronal inclusions on H&E staining that also show strong immunoreactivity for FUS, predominantly within subcortical regions [49]. Interestingly, basophilic inclusions have been noted in ALS-*FUS* without FTLD [8, 31, 80]. The considerable variation in neuropathology between patients with FTLD does not correlate clearly with differences in clinical features, compounding the difficulty in making assertions regarding neuropathological aetiology. However, there is some evidence that aFTLD-U cases show stereotypic clinical characteristics [74].

### An ‘oligogenic’ hypothesis of disease

While there might be doubt about some *FUS* variants being directly causal, it may be speculated that they play a role as part of an oligogenic susceptibility profile, with mutations occuring in more than one ALS/FTD gene in a single patient at a higher rate than would be expected by chance. This hypothesis is exemplified in lineages containing inherited mutations that display incomplete penetrance in phenotypically normal family members, where affected individuals possess risk factor variants in multiple genes. Such oligogenic variants have the potential to influence the neuropathology or phenotype of a more dominant mutation in ALS/FTD, as has been evidenced in several cases [14, 15, 86, 87]. This effect is now recognised in the context of several other diseases [6, 60, 77, 95]. Investigating the relative contribution of these modifier variants to disease pathogenesis is often challenging, requiring extensive experimental as well as computational genomic and bioinformatic analysis, and further work is required to elucidate the influence of such genetic modifiers on the disease course of FUS-associated ALS/FTD.

### Evidence for distinct pathogenesis of ALS-*FUS* and FTLD-FUS

Despite structural and functional similarities between FUS and TDP-43, they are differentially post-translationally modified (Table 2). TDP-43, for example is extensively phosphorylated and cleaved to produce toxic, aggregate-prone C-terminal fragments, while endogenous FUS is maintained at full length, even in disease [52]. The fact that neurodegeneration is induced through the presence of a single point mutation within the *FUS* gene in some cases of familial ALS, but FUS inclusions are the predominant pathological characteristic of a subset of FTLD cases *despite* lack of mutation to its corresponding gene, has led to the suggestion of each disease being driven by separate pathogenetic mechanisms. Post-translational modification of proteins can drastically alter their function; for example by changing their conformational shape through the addition of charged amino acids at certain residues. Recently, evidence has emerged involving specific post-translational modifications of FUS that suggest a possible explanation for the differences in pathogenesis between the two diseases, whilst simultaneously accounting for their shared FUS immunoreactivity.

**Table 2:**
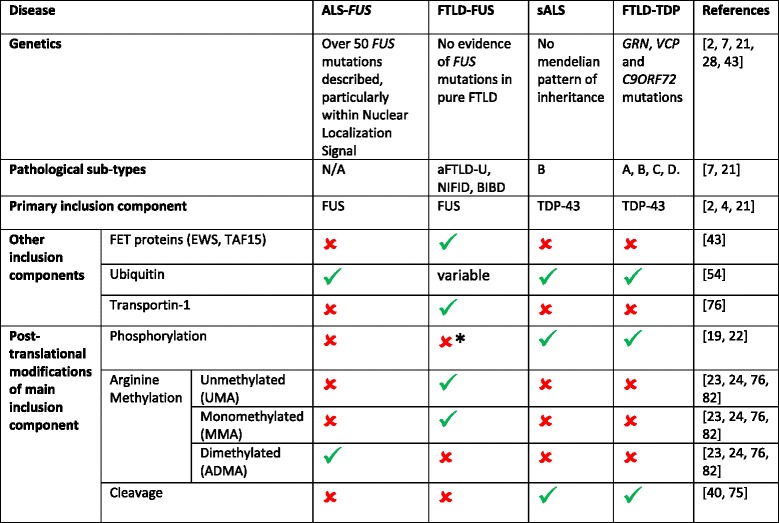
Pathogenetic differences between ALS-FUS and FTLD-FUS

Under normal physiological conditions, FUS does not appear to undergo any post-translational modifications. Arginine methylation is facilitated by protein methyltransferase 1 (PRMT1), which inhibits binding to Transportin-1 and prevents nuclear re-localization. Hypermethylation of FUS protein only occurs in ALS-FUS, and not FTLD-FUS, suggesting possible insight into the way FUS aggregates are the predominant pathological characteristic of FTLD-FUS despite the absence of causative mutations, * = FUS is phosphorylated in response to DNA damage, some evidence of DNA damage in FTLD

Arginine methylation is a common post-translational modification of RNA-binding proteins involving the addition of methyl groups either symmetrically or asymmetrically to nitrogen atoms in the arginine side chain [9]. At least 20 sites within the FUS protein, mainly located in the RGG3 domain, are arginine methylated [63], mediated primarily by protein *N*-arginine methyltransferase 1 (PRMT1) [24], inhibition of which limits the capacity of mutant FUS to localize to the cytoplasm to form inclusions [82]. The nuclear import karyopherin protein Transportin-1 strongly co-localizes with FUS in FTLD-FUS [13], however in FTLD-FUS all FET proteins co-localize with Transportin-1, whereas ALS-*FUS* inclusions contain exclusively FUS. In 2012, Dormann et al. [23] showed that arginine methylation impairs Transportin-1 dependent nuclear import of FUS by preventing Transportin-1 binding upstream of the NLS. Using a novel methyl-specific antibody, they also showed that inclusions in ALS-*FUS* are extensively asymmetrically methylated. The authors used this evidence to speculate that mislocalization of FUS in ALS is caused by mutations in the NLS that are then exacerbated by arginine methylation in the RGG3 domain, whereas mislocalization in FTLD-FUS may be caused more broadly by hypomethylation of all FET proteins, mediated by altered Transportin-1 binding. This concept has recently been elaborated through the development of monoclonal antibodies capable of distinguishing individually methylated forms of FUS protein - unmethylated arginine (UMA), monomethylated arginine (MMA) or asymmetrically arginine dimethylated (ADMA) [76]. Using these antibodies, it has been possible to show that FUS inclusions in FTLD-FUS contain UMA and MMA, whilst inclusions in ALS-*FUS* contain only ADMA. UMA and MMA show an increased binding capacity to Transportin-1, whilst arginine methylation decreases its binding capacity and thus reduces nuclear import. Together, these studies implicate a role for arginine methylation in FUSopathy, and for the first time provide substantive evidence of each disease being driven, at least in part, by distinct pathogenetic mechanisms.

Phosphorylation is another common post-translational modification involving the covalent addition of a phosphoryl group by a protein kinase. FUS is phosphorylated by DNA protein kinase (DNA-PK) in response to DNA damage, which leads to its cytoplasmic accumulation [22], and there is evidence of DNA damage in FTLD-FUS patients. Additionally, one group has shown that phosphorylation of a specific C-terminal tyrosine residue impairs Transportin binding and prevents nuclear import [19]. Given the multifactorial nature of FUSopathy (and indeed, ALS/FTD in general) it is likely that both types of post-translational modification contribute to disease pathogenesis. However, the actual pathological significance and contribution of each to human disease is still yet to be demonstrated.

### Current rodent models of ALS-*FUS*

Significant progress has been made regarding modelling FUSopathies associated with *FUS* mutations in vivo; including the development of cellular, vertebrate and invertebrate models. While invertebrate models using *Saccharomyces cerevisiae* [33], *Drosophila melanogaster* [41], and *Caenorhabditis elegans* [50] have yielded various insights, vertebrate models in general provide more translatable results to human disease because of their increased genetic homology - at the notable expense of being considerably more complex and time consuming to generate. Recently several groups have characterised their own ALS-*FUS* rodent models created using a variety of transgenic and viral-mediated methods (Table 3).

**Table 3:** Rodent models of ALS-*FUS*

Study	Species	Model type	Background	Phenotype	Neuropathology	Gene expression analysis	Other
Kino et al. [37]	Mouse	Transgenic knockout (FUS -/-)	Mixed C57BL/6-ICR	Hyperactivity, reduction in anxiety, lowered body weight. No reduction in motor activity or observation of ALS phenotypic features.	Non-progressive vacuolation of CA3 region at 8-10 weeks. No evidence of neurodegeneration	No significant enrichment of specific profiles or changes in expression of other ALS-FTD related genes	Underexpression of FUS mRNA
Robinson et al. [67]	Mouse	Transgenic, FUS gene including R522G mutation and lacking RNA recognition motif	B6CBAF1/J	Lowered body weight, early lethality, pronounced tremor around two days before death	Large cytoplasmic FUS-positive inclusions in cortex and brainstem. No evidence of neurodegeneration	Not studied.	Significant FUS overexpression
Shelkovnikova et al. [73]	Mouse	Transgenic, using human aggregate prone FUS-variant lacking Nuclear localization signal and RNA binding motif (expressed at lower levels than endogenous FUS)	Mixed C57BL/6-CBA	Severe motor dysfunction at ~3 months, death within 2 weeks of symptom onset	FUS-positive inclusions in lower motor-neuron cell bodies, some ubiquinated inclusions. Significant SC neuronal loss and neuroinflammation. Prominent muscular atrophy	Not studied.	-
Verbeeck et al. [92]	Mouse	Somatic brain transgenic using intracerebral injection of AAV incorporating either R521C, ΔR14, or WT-overexpression	B6C3F1	Healthy at time of death (3 months), no obvious motor impairment in any line	Increased cytoplasmic FUS expression in both mutants, however only some ΔR14 animals showed actual FUS-positive, ubiquinated inclusions. No evidence of neurodegeneration	Not studied.	High levels of FUS mutants within cytoplasm
Mitchell et al. [47]^a^	Mouse	Transgenic, over-expressing human WT FUS (hFUS +/+)	C57BL/6	Rapid decline in motor function from 4 weeks old, hind limb paralysis at 8 weeks	Intense perinuclear and cytoplasmic FUS staining in cortical neurons without neuronal loss. Granular cytoplasmic FUS inclusions in spinal cord with neuronal loss and astrogliosis	Not studied.	Increased nuclear and cytosolic FUS levels
Qiu et al. [61]^a^	Mouse	Transgenic expressing mutant R521C construct	C57BL/6	Severe motor dysfunction – spastic paraplegia, muscle wasting, abnormal gait etc. Death within 6 weeks of symptom onset	Significant (~50 %) loss of motor neurons with moderate astrogliosis in the spinal cord. FUS expression mainly seen in nuclei. Dendritic and synaptic defects in both SC and cortical neurons	R521C mutation causes splicing defects in genes that regulate synaptic functions. 766 genes involved in range of cellular functions identified that are differentially expressed between mutant and WT mice	FUS-R521C–associated DNA damage causes changes in downstream *bdnf* signalling
Sephton et al. [70]^a^	Mouse	Transgenic, Cre-recombinase approach. Created two lines expressing either R521G mutation or overexpressing WT FUS at low levels	C57BL/6	Both lines showed severe motor dysfunction followed by early lethality. FUS^R521G^ mice that escaped early lethality showed less pronounced motor dysfunction and deficits in spatial awareness	No FUS proteinopathy or aggregation in either line. No evidence of neuronal loss. Denervation of neuromuscular junctions and muscular atrophy in both lines	Pre-symptomatic FUS^WT^ – differential expression of 185 genes, particularly related to DNA repair and regulation of cell proliferation. No statistically significant expression changes in FUS^R521G^ mice	Reduced levels of R521C mRNA at synapses in response to mGluR activation.
Sharma et al. [71]	Mouse	Transgenic, Cre-LoxP with expression of WT human FUS, R521C or P525L mutation at *MAPT* locus	C57BL/6	Both mutant lines showed hind limb weakness (P525L more severe) with no effect on survival. No phenotype in WT line	Progressive, mutation-dependent neurodegeneration and denervation of NMJ. Large increase in cytoplasmic FUS aggregation without inclusion formation. Additional astrocytosis and microgliosis in mutant SC but not WT	Not studied.	Additional KO model demonstrates loss of FUS alone not responsible for motor phenotype
Scekic‐Zahirovic et al. [69]	Mouse	Transgenic, knock-in mice using ablation of NLS (exon 15), and knock-out (-/-) mice lines	C57BL/6	Immediate perinatal lethality of both lines from respiratory insufficiency	Cytoplasmic FUS mislocalization in transgenic line without inclusion or stress-granule formation. Knock-in line showed reduced motor neuron numbers associated with neuronal apoptosis. FUS mislocalization affected HDAC1 aggregation	353 genes differentially expressed by both lines in the same direction compared to wild-type. Both lines showed significant splicing alterations	Cross with specific Cre-line rescued FUS mislocalization but not perinatal lethality phenotype.
Huang et al. [30, 29]	Rat	Transgenic expressing mutant FUS R521C construct. Additional model overexpressing human WT FUS	Sprague Dawley	Progressive paralysis of both fore and hind limbs in R521C mutant model but not in human WT overexpressing model. Spatial awareness and memory deficits in mutant line	Ubiquitinated, diffuse cytoplasmic FUS expression and glial activation in mutant FUS model but not WT model. Hippocampal and cortical neuron loss in both models	Not studied.	-

Despite several models utilising the same R521C mutation and transgenic approach, results notably vary. Two knock-out models created before the identification of the significance of *FUS* in ALS are not included for clarity

*AAV* Adeno-associated virus, *WT* Wild-type, *SC* Spinal cord, *FUS* Fused-in-sarcoma, *MAPT* Microtubule-associated protein tau

^a^Model publicly available through Jackson Laboratories as of June 2016

Originally, *FUS* was identified for its role as a fusion oncoprotein in the development of round cell liposcarcomas and human myeloid leukaemias. Nine years before the recognition of its relevance to ALS/FTD, two groups created FUS knock out (KO) mouse models to investigate its functional role and effects of haploinsufficiency. Hicks et al. (2000) used insertional site mutagenesis to create a null mutation that effectively caused *FUS* transcriptional silencing. Mice failed to suckle, dying within 16 h of birth, and affected cells showed an increase in aneuploidy and chromosomal aberrations, which the authors used to highlight the importance of *FUS* in genomic maintenance and chromosomal stability. Another group used a similar non-functional insertional cassette technique to adequately disrupt *FUS* transcription, albeit resulting in low level expression of a severely truncated, non-functional protein [38]. Kuroda et al. analysed their model solely in terms of the reproductive system, and neither group investigated their model neuropathologically. The first neuropathological analysis of *FUS* KO mice was performed recently [37], utilising the same mice as Hicks but heterozygotes were outcrossed with ICR mice before inter-crossing the F1 progeny. *FUS*^-/-^ mice displayed a reduced body weight but no motor phenotype, and numbers of choline-acetyltransferase positive neurons were normal. Mice did display non-progressive vacuolations, particularly in the hippocampus. The lack of both motor phenotype and neurodegeneration in KO mice suggests that FUS depletion alone is insufficient to cause ALS symptoms or pathology. Interestingly however, a reduction in motor activity has been reported in two zebrafish knock-down models using anti-sense morpholino oligonucleotides [5, 34]. This is perhaps surprising given that morpholinos are generally only capable of partially reducing target gene expression. The reason for this apparent phenotypic discrepancy between species is unclear, but the difficulties in identifying subtle motor impairments in embryonic zebrafish are noted. Alternatively, it may feasibly be due to a more significant functional role played by *FUS* in the developing zebrafish embryo than the adult mouse.

The effects of mutated *FUS* displaying a gain-of-function mechanism of toxicity have been described in various cell culture experiments, and the cellular toxicity of wild-type (WT) *FUS* overexpression has been documented in yeast [33]. The aggregation propensity of wild-type (WT) FUS was investigated by Mitchell et al. (2012) in mice, who overexpressed WT human FUS cDNA under the control of a mouse prion protein gene promoter. FUS^+/+^ mice developed a rapid decline in motor function from 4 weeks old, and displayed intense FUS perinuclear inclusions in Layer V motor cortex and striatum, with additional diffuse cytoplasmic staining throughout cortical neurons, despite total FUS expression being only 1.7 times higher than non-transgenic mice. Neuronal loss was seen in the spinal cord but not in the brain, with consequent impaired neuromuscular function. Aggregation of structurally normal (i.e. non-mutation affected) FUS is characteristic of FTLD, however the severe motor dysfunction seen in these mice supports the suggestion that aggregation of WT FUS is sufficient to induce neurodegeneration and the motor phenotype of ALS.

Normal FUS protein contains several distinct functional domains, including multiple RNA binding regions, a C-terminal Zinc-finger motif and a highly-conserved Nuclear Localization Signal (NLS). Recently, several groups have attempted to drive pathology by inducing mutations in the NLS. In perhaps the most extensive characterisation of a *FUS* rodent model to date, Qiu et al [61] used a prion promoter to drive expression of the human R521C mutation in transgenic mice, leading to severe motor deficits and death within 4-6 weeks of symptom onset. While there was significant and progressive neuronal loss, *FUS* expression was predominantly nuclear, demonstrating that neurodegeneration induced by the mutation was not caused by aggregation of cytoplasmic FUS. Expression analysis identified significant alterations in genes involved in transcription and RNA processing, which were predicted to be the cause of severe dendritic and synaptic defects in spinal and cortical motor neurons. In addition, the authors identified deficiencies in DNA repair caused by R251C mutant FUS being unable to interact with the chromatin re-modelling factor HDAC1. However, this study did not utilise mice expressing human WT FUS, so it is unknown whether these defects were the result of the mutation or overexpression of FUS protein itself.

Some groups have investigated more dramatic genetic FUS alterations. Shelkovnikova et al. [73] generated transgenic mice that lacked both the entire NLS and RNA binding motifs, while still retaining the N-terminal prion-like domain. This allowed the investigation of FUS pathogenicity independent of its ability to sequester RNA binding proteins and its effect on RNA processing, while still being highly aggregate prone. Mice showed degeneration of motor neurons, neuroinflammatory reactions with abrupt development of a severe motor phenotype, death within a few days of symptom onset, and distinct FUS inclusions within LMN cell bodies and in the motor cortex. Taken together, these results are significant because they suggest that FUS aggregation and inclusion formation caused by mutant FUS is sufficient to induce neurodegeneration independently of the role of FUS in RNA metabolism. Robinson et al. [67] combined approaches, and created a model that both lacked an RNA recognition motif and contained the R522G point mutation within the NLS. Mice exhibited pronounced tremor followed by early death, and widespread cytoplasmic FUS aggregation in the cortex, brainstem and cerebellum, suggesting that lack of RNA binding to FUS increases its inherent propensity for cytoplasmic aggregation. However, there was no evidence of neuronal loss or astrogliosis. While significant as proof-of-concept, the extent of genetic alteration required to induce pathology in both studies makes the relationship to human disease pathogenesis uncertain.

A different approach has been to use somatic brain transgenesis (SBT) to overexpress human mutant *FUS* cDNA [92]. Mice were intracerebally injected with an adeno-associated virus vector, incorporating either the R521C mutation or FUS lacking the nuclear localization signal entirely (∆14). The advantage of this method is its speed - mice can be generated within a few months as opposed to years when using traditional transgenic approaches. Affected mice however showed no phenotype when euthanized at 3 months. Neuropathologically, FUS R521C mice showed a large increase in cytoplasmic FUS without obvious NCI formation or neurodegeneration, while FUS ∆14 mice displayed FUS pathology more closely mimicking human disease including ubiquitin/p62 positive NCI. Additionally, the authors also used this method to overexpress wild-type (WT) human FUS. Unlike those reported previously [47], overexpression of WT FUS did not cause any abnormal pathology or neurodegeneration. Huang et al. [30] created two rat models; one expressing the R521C mutation, and one overexpressing WT human FUS at comparable levels. Similar to their mice counterparts, R521C rats developed a progressive paralysis resembling human ALS, and also displayed motor neuron axonopathy with motor neuron loss in both the hippocampus and the cortex. NCI were absent, but diffuse cytoplasmic FUS staining was noted in ventral horn motor neurons. Rats overexpressing WT FUS did not develop a significant motor phenotype, but did display deficits in spatial learning and memory, with moderate neuronal loss in the frontal cortex. Consistent with previous studies [47] this suggests that overexpression of WT FUS is sufficient to induce neuronal loss but that mutant FUS is more toxic than WT. The phenotypic differences between the Verbeeck model and other models involving the same mutations/WT-overexpression may be due to the transient expression of the adenovirus before it is cleared, the localized expression of AAV transgenes, or because mice were euthanized before the development of severe NCI formation/phenotype.

Finally, Sephton et al. [70] used a Cre-inducible transgenic approach to create two mice lines, expressing either human WT FUS (FUS^WT^) or the R521G (FUS^R521G^) mutation, both at comparably low levels. Nearly all mice developed a severe motor phenotype and early lethality, with some FUS^R521G^ mice escaping this and exhibiting subtle motor impairments and altered sociability. This is in contrast to previous studies mentioned above, which concluded that expression of WT FUS is less pathogenic than mutant. While there was no evidence of FUS cytoplasmic mislocalization, aggregation or neuronal loss in either model, the authors suggested these features are end-stage pathological markers of human disease, precipitated by the denervation of neuromuscular junctions (NMJ) that caused the motor phenotype. Interestingly, differences in gene expression between models suggests that FUS^WT^ exhibits a loss-of-function mechanism through its effects on gene expression, while FUS^R521G^ exhibits gain-of-function toxicity through its disruption of synaptic homeostasis. This is consistent with the effects of the R521C mutation on the regulation of genes relating to synaptic function in the Qiu et al. model. Most recently in an elegant study, Sharma et al. [71] used Cre-LoxP to generate mice expressing a single copy of human WT FUS, (FUS^WT^), R521C (FUS^R521C^) or P525L (FUS^P525L^) at the *MAPT* locus, and an additional knock-out model. As in previous over-expression models [31, 47], they demonstrated that overexpression of WT FUS alone was sufficient to induce neurodegeneration, but that mutant FUS is more stable and pathogenic than WT. Pathogenicity in these models was mutation dependent, with FUS^P525L^ mice showing motor neuron specific degeneration at a younger age and a greater degree of cytoplasmic FUS mislocalization than in the FUS^R521C^ model, reflecting the mutation-dependent phenotype of human cases bearing these mutations [8, 48]. As in the previous Cre-LoxP model [70] there was also significant denervation of NMJ in both mutant models preceding neurodegeneration, adding weight to the suggestion that neuronal loss is a downstream consequence of NMJ denervation caused by these mutations. However, the survival time of these mice was normal, and there was no NCI formation in any line.

## Conclusion

Given its significance in the pathophysiology of ALS/FTD, modelling FUSopathy in vivo has been a focus for several groups. However, reproducing both the motor dysfunction phenotype and the distinct neuropathological features of *FUS*-linked ALS has proven challenging in rodents. In particular, while the models discussed here provide clues as to the pathomechanistic role of FUS and its significance in the neurodegeneration of human disease, none of the above completely recapitulate the features of human ALS, and all current models compromise in at least one area of human pathophysiology. To our knowledge there is currently no vertebrate model that mimics the unique post-translational modifications associated with human FTLD-FUS, which are clearly distinct from FUSopathy with *FUS* mutations.

Several of the models described above were created using traditional plasmid-mediated transgenic methods, which have their own methodological limitations. Small cDNA-based transgenes lack the regulatory upstream sequences found as part of many complex mammalian genes. Also, several of the models described here use heterologous promoter sequences that cause expression of the transgene in excess of what would normally be expected in human disease. A Bacterial Artificial Chromosome (BAC) approach may somewhat ameliorate these issues, by allowing the integration of important regulatory sequences some distance upstream of *FUS* in addition to an endogenous mouse promoter, as well as being modifiable to direct cell-type specific gene expression. Unlike small cDNA-based transgenes, the expression of BAC clones also correlates closely with copy number. This approach has already been used in the context of ALS to generate two *C9ORF72* mouse models [55, 57], and we are currently in the process of characterising our own *FUS* model that has been created in such a way. Additionally, the much publicised CRISPR-Cas9 genome editing tool is reaching stages of development that allows for the knock-in of specific point mutations, without extensive off target effects or the need for exogenous regulatory sequences, and it is anticipated that this tool will be used extensively in neurodegenerative disease modelling in the coming years. Finally, of note is that many of the aforementioned studies did not conduct significant transcriptional profiling of their models, which is perhaps surprising given the known role of FUS in the expression regulation of several target genes identified in cell culture experiments. Elucidating the precise normal physiological function of *FUS* and further refinement of vertebrate models will likely aid our understanding of its role in the pathogenesis of both ALS and FTD.

## Abbreviations

ALS, Amyotrophic lateral sclerosis; bvFTD, Behavioural variant FTD; FTD, Frontotemporal dementia; FTLD, Frontotemporal lobar degeneration; FUS, Fused-in-sarcoma; LMN, Lower motor neuron; MND, Motor neurone disease; PNFA, Progressive non-fluent aphasia; SD, Semantic dementia; UMN, Upper motor neuron
